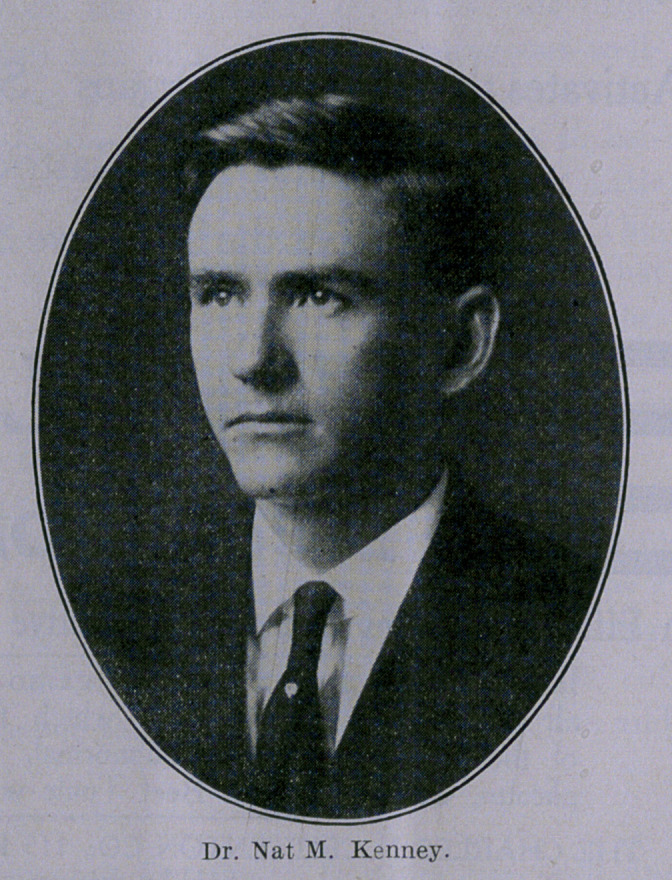# Editorialets

**Published:** 1910-10

**Authors:** 


### Editorialets.

---

WE REGRET to announce the death of Dr. George F. Perry, of Hamilton, Texas; of Washington University, St. Louis, 1875; of Ochiltree; a member of the State Medical Association of Texas; in 1890, Representative in the State Legislature. He died in St. Francis Hospital, Colorado Springs, August 30th, from uremia, aged 63.

---

THE ASSOCIATION OF TEXAS HEALTH OFFICERS held a largely attended meeting at Houston, September 26th and 27th (ult.), under the presidency of Dr. Brumby. Report received too late for this issue.

---

THE SEVENTH COUNCILOR DISTRICT MEDICAL SOCIETY held its regular meeting in Austin, September 22d (ult.). The papers read are, *by law*, the property of the *State Journal of Medicine*. There were some thirty members present. Dr. M. L. Graves, of Galveston, who was down for an illustrated lecture on diseases of the chest, failed to be present; a great disappointment to the members, many of whom came miles to hear him.

---

TIT FOR TAT.—A *reductio ad absurdum*. The little publication of the National Association of Retail Druggists has raised a tempest in a teapot. It proposes a propaganda to get a law passed to prevent doctors from dispensing medicine! The claim is that the law will not allow a druggist to diagnose and prescribe (practice medicine); therefore, it should not allow a doctor to give a patient medicine!

---

MUCH ADO ABOUT NOTHING.—Mr. Abraham Flexner has surely "started something." He has stirred up a hornet's nest and gotten the medical colleges to howling. No thief ever felt the halter draw and entertained a good opinion of the law, or words to that effect. Mr. Flexner rather overdid it; like the preacher who prayed for rain, and got hail. Everybody recognizes the fact that there are too many medical schools; that the requirements in many are too feeble, graduation too easy, equipment for teaching in some appear only on paper, and that half-cooked doctors are turned loose on a too confiding public, largely in excess of the demand. This is an evil that calls for reform; but Mr. Flexner's report has not the effect of law, and I don't see why the colleges that are hit should get so hot about it. His criticism does not amount to a row of pins; for instance, amongst the five or six colleges commended by Mr. Flexner as filling the requirements—his ideal—he mentions the Medical Department of the University of Texas, and puts it in the class with Johns Hopkins, Pennsylvania University, Ann Arbor et al. Now, it is notorious that our own Medical Department of the University of Texas—the pride of the State, while efficient and ably officered, turning out well-qualified young doctors—*has no laboratory equipment worth mentioning.* And our wise Governor cut off an appropriation to better it. The late Professor McLaughlin extemporized a little chebang in his bedroom—in which he made his researches. This defect has caused nearly every pathologist who has come to that college to teach, to quit and go elsewhere. Dr. Austin left because (he told me) he couldn't work without tools. Allen J. Smith, Thayer, and, away back, Geo. Dock, all took positions where there was equipment.

Now, I have no doubt Abraham means well. But he rather overdid it, as I said. It reminds me of the boy who, desiring to cut off his puppy's tail, got his daddy to stretch the tail across a stick of wood. Just as the axe descended, daddy pulled the dog, and sonny cut his pup's tail off—just back of the ears. Abe cut off too much tail. It was all tail, in his opinion. Only nose and ears left.

---

THE handsome and talented Dr. Oscar Dowling has been appointed President of the Louisiana State Board of Health.

THE FIRST NORTHER.—

Now fades the glimmering straw hat out of sight,
And all the clothes a wintry aspect wear,
Save where the editor, always in a tight,—
Must wear his linen duster still, or go bare.

Please remit.

## Figures and Tables

**Figure f1:**
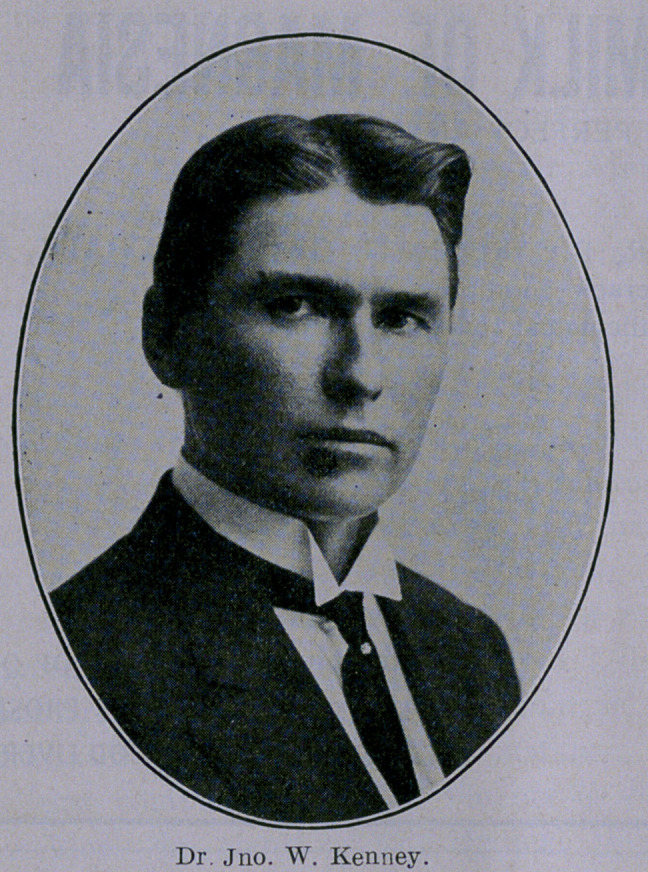


**Figure f2:**